# Epidemiologic study of in-hospital cardiopulmonary resuscitation among pediatric patients: A retrospective, population-based cohort study in South Korea

**DOI:** 10.1097/MD.0000000000030445

**Published:** 2022-09-09

**Authors:** Tak Kyu Oh, Chang Won Choi, In-Ae Song

**Affiliations:** a Department of Anesthesiology and Pain Medicine, Seoul National University Bundang Hospital, Seongnam, South Korea; b Department of Anesthesiology and Pain Medicine, College of Medicine, Seoul National University, Seoul, South Korea; c Department of Pediatrics, Seoul National University Bundang Hospital, Seongnam, Republic of Korea; d Department of Pediatrics, Seoul National University College of Medicine, Seoul, Republic of Korea.

**Keywords:** cardiopulmonary resuscitation, child, cohort studies, mortality, resuscitation

## Abstract

We aimed to examine the clinical trends of in-hospital cardiopulmonary resuscitation (ICPR) and factors associated with live discharge following ICPR. As a national population-based cohort study, data were extracted from the South Korean National Inpatient Database. This study included 8992 pediatric patients under 18 years of age who underwent ICPR due to in-hospital cardiac arrest during hospitalization in South Korea between 2010 and 2019 (10 years). The annual prevalence, survival, duration of hospitalization, and total cost of hospitalization at ICPR were examined as clinical trends. In 2010, 7.94 per 100,000 pediatric patients received ICPR; the prevalence increased to 11.51 per 100,000 pediatric patients in 2019. The 10-year survival rates were similar, and the in-hospital, 6-month, and 1-year survival rates over 10 years were 44.0%, 34.0%, and 32.4%, respectively. The mean length of hospital stay at ICPR in 2010 was 20.7 (95% confidence interval [CI]: 19.3–22.2) days; this decreased to 16.6 (95% CI: 15.2–18.0) days in 2019. The mean total cost at ICPR was 11,081.1 (95% CI: 10,216.2–11,946.1) United States Dollars (USD) in 2010; this increased to 22,629.4 (95% CI: 20,588.3–24,670.5) USD in 2019. The prevalence of ICPR increased among pediatric patients in South Korea between 2010 and 2019; however, the survival rates were similar for the 10 years. The length of hospital stay at ICPR gradually decreased from 2010 through 2019, while the total cost of hospitalization at ICPR has gradually increased between 2010 and 2019.

## 1. Introduction

Cardiopulmonary resuscitation (CPR) is a life-saving procedure performed on patients suffering from cardiac arrest.^[1]^ In-hospital CPR (ICPR) is performed in patients who experience in-hospital cardiac arrest (IHCA) during hospitalization.^[2]^ Unlike in adults, IHCA is a rare event in pediatric patients.^[3]^

In the United States, 15,200 pediatric IHCA events were reported annually between 2008 and 2017.^[4,5]^ Jarrod et al^[6]^ reported that based on the Kids’ Inpatient Database of the United States, ICPR was performed in 5807 children, with a prevalence of 0.77 per 1000 hospital admissions. Moreover, they reported that in-hospital mortality following ICPR among pediatric patients was 51.8% and greater among patients aged ≥1 year (68%) versus <1 year (44%).^[6]^ Since the characterization of pediatric ICPR differs from that of adult ICPR,^[5]^ early risk identification, prevention, delivery of high-quality CPR, and post-ICPR care is crucial for pediatric patients.^[7]^ However, information regarding pediatric ICPR is still lacking.

Therefore, using the nationwide registration database of South Korea, we aimed to examine the clinical trends of ICPR and associated factors for live discharge following ICPR in South Korea. The annual prevalence, short- and long-term survival, duration of hospitalization, and total cost of hospitalization at ICPR were examined as clinical trends.

## 2. Materials and Methods

### 2.1. Study design, setting, and ethical statement

For this nationwide, population-based cohort study, the Strengthening of the Reporting of Observational Studies in Epidemiology guidelines were followed.^[8]^ The study protocol was approved by the Institutional Review Board of Seoul National University Bundang Hospital (IRB number: X-2011-651-901), and the National Health Insurance Service (NHIS) permitted data sharing following the approval of the study protocol (NHIS-2021-1-266). The requirement for informed consent was waived by the Institutional Review Board as anonymized data were used in this study.

### 2.2. Data source and study population

The NHIS database was used for this study. As the sole public insurance database system in South Korea, the NHIS database contains information on all drug prescriptions, disease diagnoses, and/or procedures. These registrations enable patients to receive financial support from the government for treatment expenses. The International Statistical Classification of Disease and Related Health Problems, 10th Revision (ICD-10) codes were used to diagnose the diseases. This study included 8992 pediatric patients aged under 18 years who underwent ICPR due to IHCA during hospitalization in South Korea between 2010 and 2019 (10 years). The data were recorded and extracted from January 1 to December 31 of each year between 2010 and 2019. Prescription codes for CPR during hospitalization among pediatric patients were used to extract the study population. The accurate death dates for all the included pediatric patients were extracted and collected until April 30, 2021.

### 2.3. Collected information

Age and sex were collected as physical information. The pediatric patients were divided into 5 groups: 1-, 2 to 5-, 6 to 9-, 10 to 13-, and 14 to 17-year-olds. As many pediatric patients underwent ICPR in the neonate period (1-year-old), patients older than 1 year were equally divided into groups by 4-year intervals. Residents at the time of ICPR were extracted and divided into 2 groups: urban areas (Seoul and other metropolitan cities) and rural areas (all other areas). Household income levels were collected and considered as covariates. All pediatric patients were divided into 4 groups based on quartile ratios according to household income level. Underlying congenital malformations were extracted using the ICD-10 codes Q00 to Q99. The duration of ICPR was collected and classified into 5 groups: <15 minutes, 15 to 30 minutes, 30 to 45 minutes, 45 to 60 minutes, and > 60 minutes. The total length of hospital stay (LOS, days) and total hospitalization costs (United States Dollar, USD) were collected. The LOS was defined as the overall duration of hospitalization from the date of hospital admission, to hospital discharge or death. Similarly, the total cost of hospitalization was calculated from the date of hospital admission, to hospital discharge or death. The results of treatment at ICPR were collected and classified into 4 groups: discharge and same hospital follow-up, transfer to long-term facility care center, death during hospitalization following ICPR, and discharge and other outpatient clinic follow-up.

### 2.4. Study objectives

First, we examined the prevalence of ICPR among pediatric patients between 2010 and 2019. The prevalence of ICPR was calculated as the total number of annual pediatric ICPR cases divided by the total pediatric population in that year. The total pediatric population was obtained from the Statistics Korea database (http://kostat.go.kr/portal/eng/index.action). Second, the in-hospital, 6-month, and 1-year survival rates from 2010 to 2019 were examined. Third, the trend regarding the total costs of hospitalization at the ICPR and LOS were examined. Fourth, the factors associated with live discharge following ICPR were examined.

### 2.5. Statistical analysis

The clinicopathological characteristics of the pediatric population were presented as mean values with standard deviation for continuous variables, and numbers with percentages for categorical variables. We created a multivariable logistic regression model to determine the live discharge rate among pediatric patients who underwent ICPR. All covariates were included in the multivariable model, and the results were presented as adjusted odds ratios (aORs) with 95% confidence intervals (CIs). The goodness of fit in the multivariable model was confirmed using the Hosmer–Lemeshow test, and no multicollinearity was observed between variables with criteria of variance inflation factors <2.0. All statistical analyses were performed using SPSS software for Windows (Version 25.0; IBM Corp., Armonk, NY, USA), and statistical significance was set at *P* < .05.

## 3. Results

**Figure 1.**
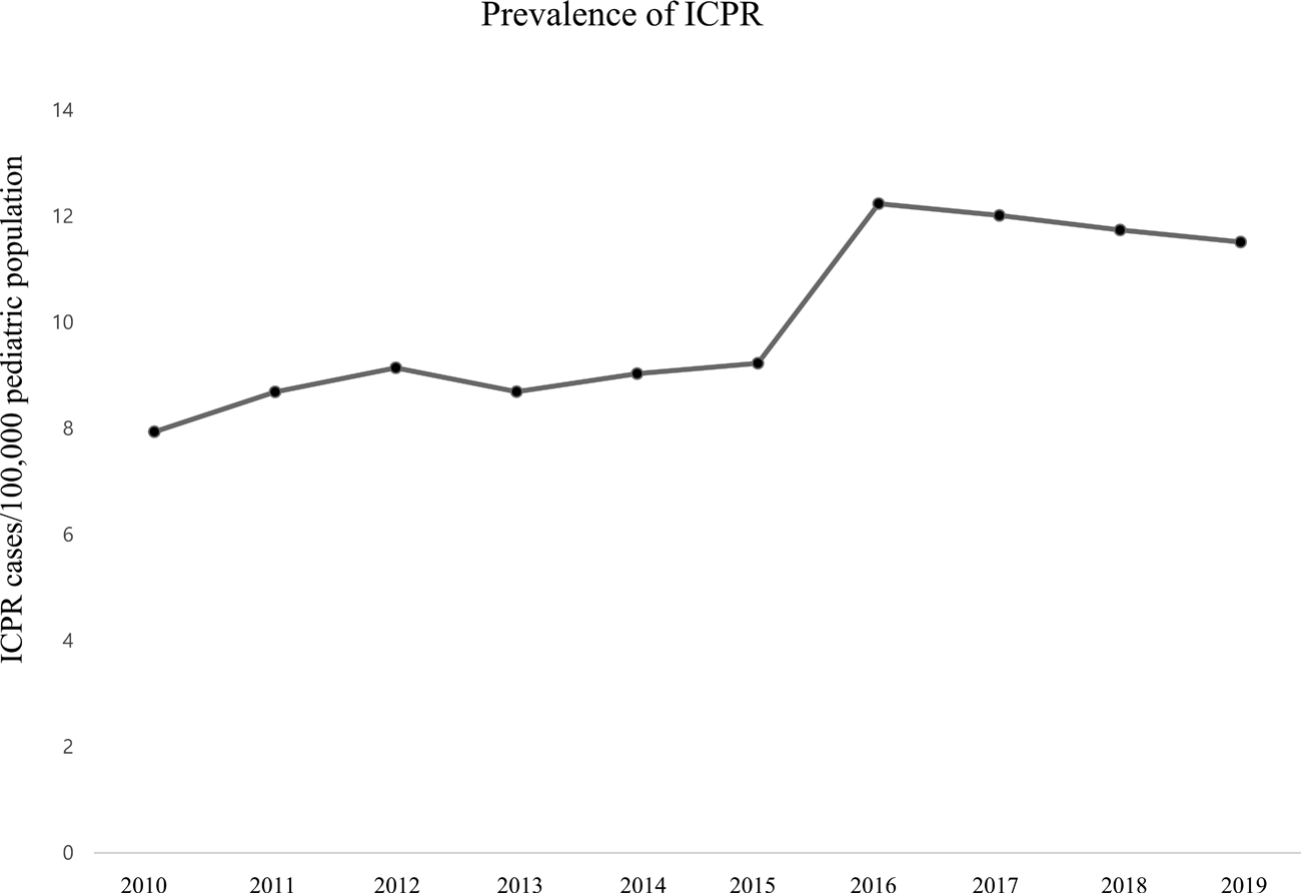
Prevalence of in-hospital cardiopulmonary resuscitation (ICPR) in South Korea among the pediatric population between 2010 and 2019.

**Figure 2.**
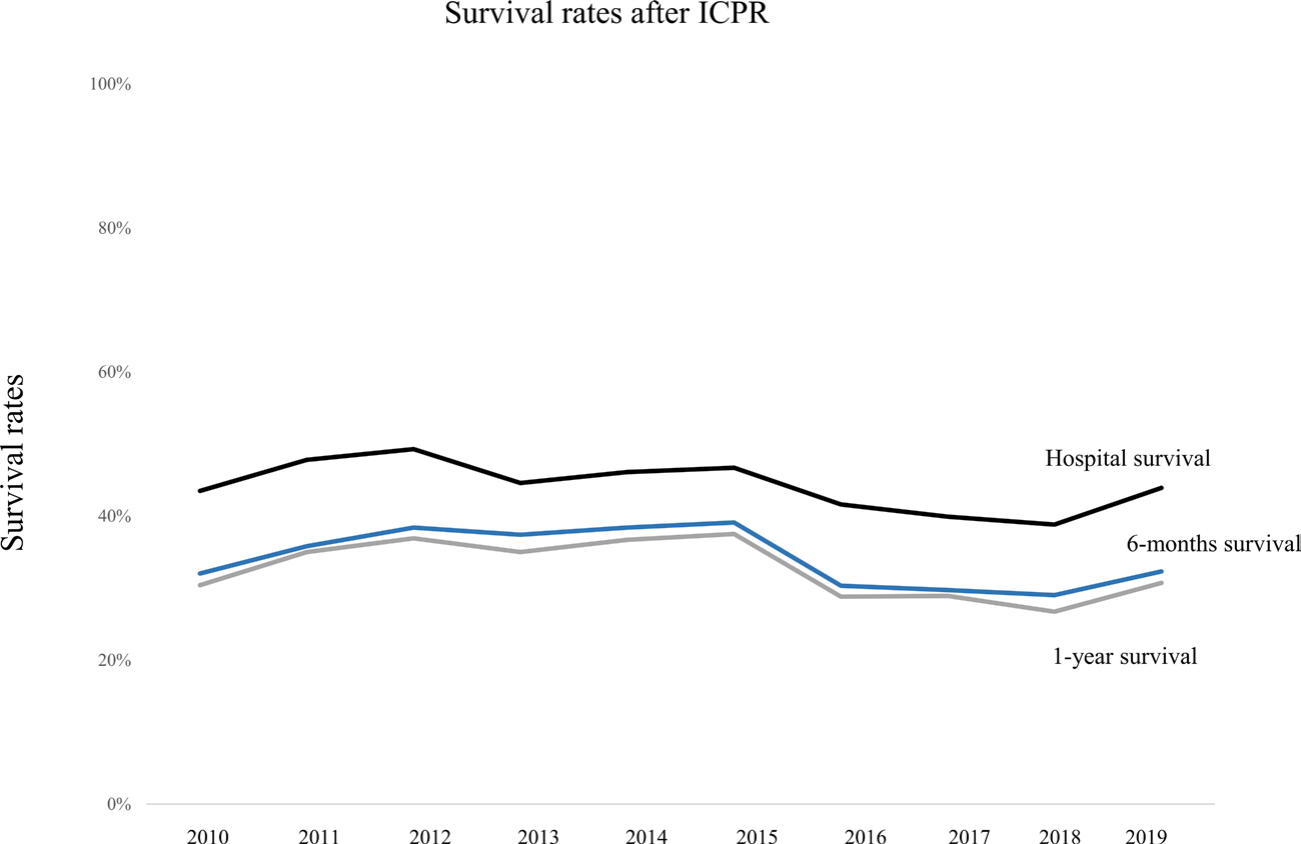
Survival rates following in-hospital cardiopulmonary resuscitation (ICPR) among pediatric patients between 2010 and 2019.

Figure 1 and Table S1, Supplemental Digital Content 1, http://links.lww.com/MD/H255 show the prevalence of ICPR in South Korea among pediatric populations from 2010 to 2019. In 2010, 7.94 per 100,000 pediatric patients experienced ICPR; the prevalence increased to 12.24 per 100,000 pediatric patients in 2016, and slightly decreased to 11.51 per 100,000 pediatric patients in 2019. Figure 2 and Table S2, Supplemental Digital Content 2, http://links.lww.com/MD/H256 show the survival rates among pediatric patients following ICPR from 2010 to 2019. The in-hospital, 6-month, and 1-year survival rates were 43.5%, 32.0%, and 30.4%, respectively, in 2010. The survival rates remained similar for 10 years; thus, the in-hospital, 6-month, and 1-year survival rates were 43.9%, 32.3%, and 30.7%, respectively, in 2019. Moreover, the overall in-hospital, 6-month, and 1-year survival rates over 10 years were 44.0%, 34.0%, and 32.4%, respectively.

**Figure 3.**
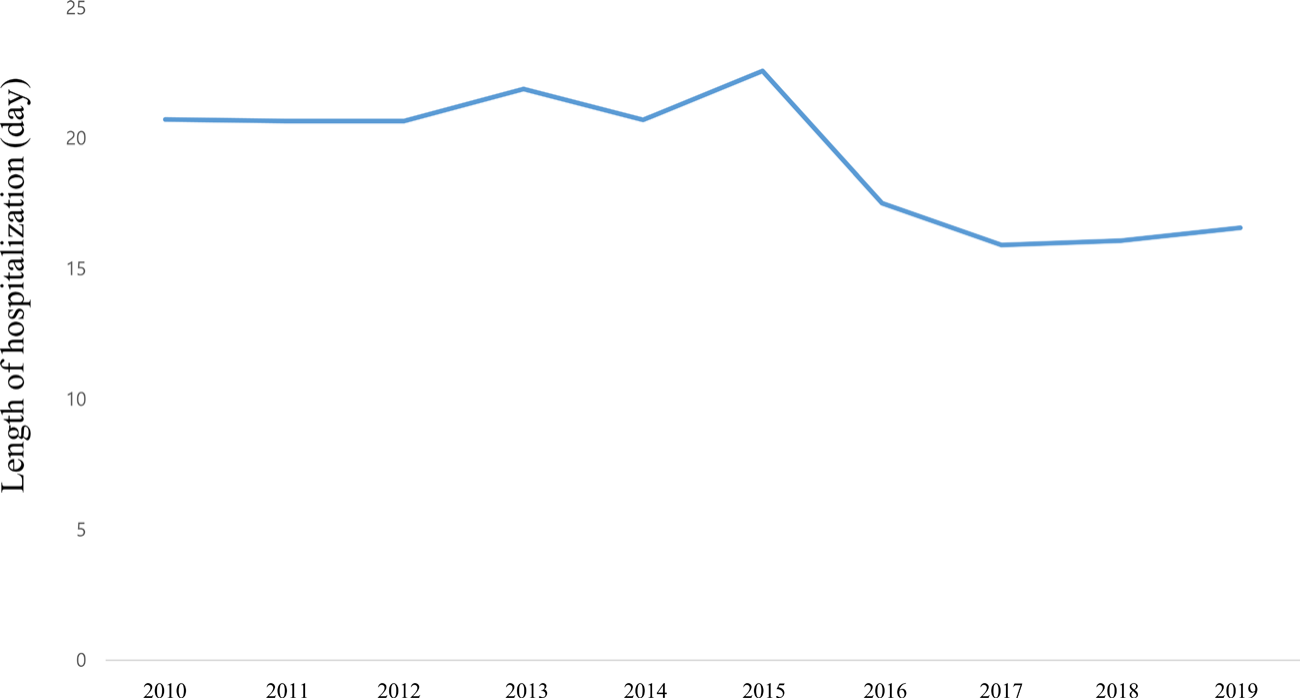
Length of hospital stay (LOS) trends at in-hospital cardiopulmonary resuscitation (ICPR) between 2010 and 2019.

**Figure 4.**
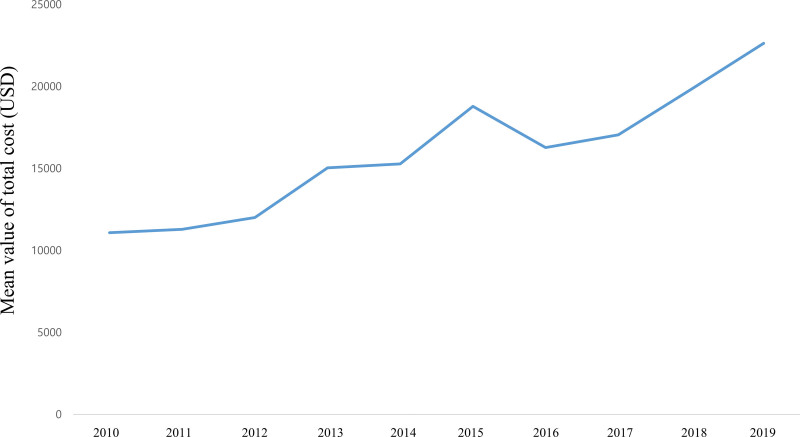
Trends of total cost for hospitalization at in-hospital cardiopulmonary resuscitation (ICPR).

Figure 3 and Table S3, Supplemental Digital Content 3, http://links.lww.com/MD/H257 (which shows the length of hospitalization) show the LOS trends at ICPR from 2010 to 2019. The mean LOS at ICPR during the 10-year period was 16.6 (95% CI: 15.2–18.0) days. Specifically, the mean LOS at ICPR in 2010 was 20.7 (95% CI: 19.3–22.2) days, decreasing to 16.6 (95% CI: 15.2–18.0) days in 2019. Figure 4 and Table S4, Supplemental Digital Content 4, http://links.lww.com/MD/H258 show the trends for the total cost of hospitalization at ICPR. The mean total cost at ICPR over 10-years was 16,049.0 (95% CI: 15,617.2–24,670.5) USD. Specifically, the mean total cost at ICPR was 11,081.1 (95% CI: 10,216.2, 11,946.1) USD in 2010, and increased to 22,629.4 (95% CI: 20,588.3–24,670.5) USD in 2019.

Table S5, Supplemental Digital Content 5, http://links.lww.com/MD/H259 shows the clinicopathological characteristics of all pediatric patients for 10 years. A total of 51.9% (4670/8992) of 1-year-old patients experienced ICPR, while 25.8% (2324/8991) of 2 to 5-year-old patients experienced ICPR. Table 1 shows the results of the multivariable logistic regression model for live discharge after ICPR among pediatric patients. Compared with the 1-year-old group, the 2 to 5 year-old (aOR: 0.82, 95% CI: 0.73–0.92; *P* = .001), 6 to 9-year-old (aOR: 0.62, 95% CI: 0.50–0.76; *P* < .001), 10 to 13-year-old (aOR: 0.68, 95% CI: 0.54–0.85; *P* = .001), and 14 to 17-year-old (aOR: 0.52, 95% CI: 0.44–0.63; *P* < .001) groups demonstrated a lower likelihood of live discharge. Compared with Q1 of the household income group, Q3 (aOR: 1.19, 95% CI: 1.02–1.38; *P* = .027) and Q4 (aOR: 1.27, 95% CI: 1.09–1.48; *P* = .003) of the household income group exhibited higher odds of live discharge. Compared with the <15 minutes ICPR duration group, the 15 to 30 minutes (aOR: 0.20, 95% CI: 0.18–0.23; *P* < .001), 30 to 45 minutes (aOR: 0.11, 95% CI: 0.09–0.13; *P* < .001), 45 to 60 minutes (aOR: 0.09, 95% CI: 0.07–0.10; *P* < .001), and >60 minutes (aOR: 0.07, 95% CI: 0.06–0.08; *P* < .001) ICPR duration groups demonstrated lower odds of live discharge.

**Table 1:** Multivariable logistic regression model for live discharge after ICPR among pediatric patients.

Variable	OR (95% CI)	*P* value
Age, yr		
1	1	
2–5	0.82 (0.73, 0.92)	.001
6–9	0.62 (0.50, 0.76)	<.001
10–13	0.68 (0.54, 0.85)	.001
14–17	0.52 (0.44, 0.63)	<.001
Sex, male	1.09 (0.99, 1.21)	.079
Residence at ICPR		
Urban area	1	
Rural area	0.90 (0.82, 1.00)	.048
Household income level		
Q1	1	
Q2	1.17 (0.99, 1.38)	.071
Q3	1.19 (1.02, 1.38)	.027
Q4	1.27 (1.09, 1.48)	.003
Unknown	1.06 (0.81, 1.39)	.660
Underlying congenital malformation	1.27 (1.13, 1.43)	<.001
Duration of ICPR		
<15 min	1	
15–30	0.20 (0.18, 0.23)	<.001
30–45	0.11 (0.09, 0.13)	<.001
45–60	0.09 (0.07, 0.10)	<.001
>60	0.07 (0.06, 0.08)	<.001
Year of ICPR		
2010	1	
2011	1.11 (0.88, 1.40)	.375
2012	1.17 (0.93, 1.47)	.176
2013	0.90 (0.72, 1.14)	.381
2014	0.95 (0.75, 1.20)	.653
2015	0.94 (0.74, 1.18)	.586
2016	0.84 (0.67, 1.04)	.113
2017	0.78 (0.63, 0.98)	.031
2018	0.74 (0.59, 0.92)	.008
2019	0.89 (0.71, 1.11)	<.001

CI = confidence interval, ICPR = in-hospital cardiopulmonary resuscitation, OR = odds ratio.

## 4. Discussion

This study demonstrated that the prevalence of ICPR increased among pediatric patients in South Korea between 2010 and 2019. The survival rates remained similar for 10 years, and the average in-hospital, 6-month, and 1-year survival rates over 10 years were 44.0%, 34.0%, and 32.4%, respectively. The LOS at ICPR gradually decreased from 2010 through 2019, while the total cost of hospitalization at ICPR gradually increased. Moreover, in the multivariable logistic regression, an age >1 year (vs 1-year-old), lower household income level, and longer duration of ICPR were associated with lower live discharge rates among pediatric patients.

Compared with the 1-year-old group, the older age group (>1 year) showed lower odds of live discharge following ICPR in this study. These results were similar to those of a previous study conducted in the United States.^[6]^ The 1-year-old group included neonatal ICPR cases, which affected the survival outcomes of the pediatric CPR cases. When a preterm or high-risk neonate is born in the delivery room, neonatologists usually perform ICPR according to the neonatal resuscitation program in South Korea.^[9,10]^ Recent studies reported that approximately one-third of infants who received delivery room CPR survive to hospital discharge^[11,12]^; the live discharge rate was higher (44%) among pediatric patients in our study. Conversely, pediatric patients aged over 1-year experienced ICPR due to various fatal diseases, such as respiratory failure or shock.^[7]^ Therefore, the survival outcomes of the 1-year-old group were better than those of the >1-year-old group.

Although the LOS at ICPR decreased, the total cost of hospitalization increased from 2010 to 2019 among pediatric patients. Therefore, the financial burden of ICPR among guardians of pediatric patients is a crucial issue. There is a lack of information regarding the financial burden among guardians of pediatric patients in the literature. A previous study reported that the average inpatient charge was 10,667 USD per pediatric patient for those who died, and 100,000 USD for those who were discharged following out-of-hospital cardiopulmonary arrest in the United States.^[13]^ However, the financial burden for guardians of pediatric patients following ICPR has not yet been identified.

The impact of household income level on outcomes following ICPR among pediatric patients was significant in this study. Since most pediatric patients did not possess an income, the household income levels of pediatric patients were determined by their guardians. Two factors may have affected this result; first, guardians with higher household income levels may exhibit higher adherence to treatment in the hospital, or more active treatment. A previous study reported that children from lower income households with chronic conditions had poorer health than those from higher income households.^[14]^ Moreover, it was reported that the familial social background of a child significantly influenced health outcomes in Germany.^[15]^ Additionally, pediatric patients usually receive expensive treatment following ICPR, such as extracorporeal membrane oxygenation or mechanical ventilatory support.^[16]^ Second, pediatric patients with a higher household income might have better accessibility to high-level medical institutions, such as tertiary academic hospitals. As parents with high incomes may live in high-income areas (Seoul, metropolitan areas, or large cities), the survival rate is typically high upon promptly receiving high-quality ICPR at high-quality medical institutions. Moreover, pediatric patients living in rural areas were associated with a 10% lower live discharge rate than pediatric patients living in urban areas (Table 1). Therefore, the survival outcome of pediatric patients might be influenced by both the socioeconomic status of their guardians, and accessibility to high-level medical institutions.

This study has several limitations. First, we could not assess information regarding important outcomes, such as the return of spontaneous circulation following ICPR, due to the lack of ICD-10 codes in the NHIS database. Second, the NHIS database lacks important information, such as alcohol consumption, body mass index, and smoking status. Third, there may be residual and unmeasured confounders in our survival analysis of pediatric patients who received ICPR. Fourth, important outcome parameters, such as neurologic sequalae after ICPR, was not evaluated in this study due to a lack of information in NHIS database. Last, the results of our study may have limitations regarding generalizability, as the clinical practice of pediatric ICPR may be influenced by the different cultures and healthcare systems of different countries.

## 5. Conclusions

In conclusion, the prevalence of ICPR increased among pediatric patients in South Korea between 2010 and 2019. The survival rates remained similar for 10 years, and the in-hospital, 6-month, and 1-year survival rates over 10 years were 44.0%, 34.0%, and 32.4%, respectively. The LOS at ICPR gradually decreased from 2010 through 2019, while the total cost of hospitalization at ICPR gradually increased between 2010 and 2019. Moreover, an age over 1-year (vs 1-year-old), lower household income level, and longer duration of ICPR were associated with lower live discharge rates among pediatric patients. This was the first study to demonstrate the change in the trend of ICPR among pediatric patients using recent national registration data.

## Author contributions

**Conceptualization:** Tak Kyu Oh, Chang Won Choi, In-Ae Song.

**Data curation:** Chang Won Choi.

**Formal analysis:** Tak Kyu Oh, In-Ae Song.

**Methodology:** Tak Kyu Oh, In-Ae Song.

**Project administration:** In-Ae Song.

**Writing – original draft:** Tak Kyu Oh.

**Writing – review & editing:** Chang Won Choi, In-Ae Song.

## Supplementary Material


