# Supplementary material for: Epidemiologic study of in-hospital cardiopulmonary resuscitation among pediatric patients: A retrospective, population-based cohort study in South Korea
Source: Medicine (Baltimore). 2022 Sep 9;101(36):e30445. doi: 10.1097/MD.0000000000030445 (PMC10980375; doi:10.1097/MD.0000000000030445)
Supplement: Supplementary file 1 [file medi-101-e30445-s001.pdf]

Supplemental digital content 1. Prevalence of ICPR in South Korea among the pediatric patients from 2010 to 2019.

Year	2010	2011	2012	2013	2014	2015	2016	2017	2018	2019
ICPR cases	806	862	886	820	830	827	1069	1019	960	913
Population	10156455	9921012	9691876	9431699	9186841	8961805	8736051	8480447	8176335	7928907
Prevalence per 10,0000	7.94	8.69	9.14	8.69	9.03	9.23	12.24	12.01	11.74	11.51

ICPR, In-hospital cardiopulmonary resuscitation
