# Supplementary material for: Epidemiologic study of in-hospital cardiopulmonary resuscitation among pediatric patients: A retrospective, population-based cohort study in South Korea
Source: Medicine (Baltimore). 2022 Sep 9;101(36):e30445. doi: 10.1097/MD.0000000000030445 (PMC10980375; doi:10.1097/MD.0000000000030445)
Supplement: Supplementary file 2 [file medi-101-e30445-s002.pdf]

Supplemental digital content 2. Mortality rates after ICPR from 2010 to 2019

	2010	2011	2012	2013	2014	2015	2016	2017	2018	2019
Hospital survival	43.50%	47.80%	49.30%	44.60%	46.10%	46.70%	41.60%	39.90%	38.80%	43.90%
6-month survival	32.00%	35.80%	38.40%	37.40%	38.40%	39.10%	30.30%	29.70%	29.00%	32.30%
1-year survival	30.40%	35.00%	36.90%	35.00%	36.70%	37.50%	28.80%	28.90%	26.70%	30.70%

ICPR, in-hospital cardiopulmonary resuscitation
