# Supplementary material for: Epidemiologic study of in-hospital cardiopulmonary resuscitation among pediatric patients: A retrospective, population-based cohort study in South Korea
Source: Medicine (Baltimore). 2022 Sep 9;101(36):e30445. doi: 10.1097/MD.0000000000030445 (PMC10980375; doi:10.1097/MD.0000000000030445)
Supplement: Supplementary file 3 [file medi-101-e30445-s003.pdf]

Supplemental digital content 3. Mean value of length of hospitalization at ICPR from 2010 to 2019

	2010	2011	2012	2013	2014	2015	2016	2017	2018	2019
LOS	20.72	20.66	20.66	21.89	20.71	22.58	17.52	15.91	16.08	16.57

ICPR, in-hospital cardiopulmonary resuscitation; LOS, length of hospitalization
