# Supplementary material for: Epidemiologic study of in-hospital cardiopulmonary resuscitation among pediatric patients: A retrospective, population-based cohort study in South Korea
Source: Medicine (Baltimore). 2022 Sep 9;101(36):e30445. doi: 10.1097/MD.0000000000030445 (PMC10980375; doi:10.1097/MD.0000000000030445)
Supplement: Supplementary file 5 [file medi-101-e30445-s005.pdf]

Supplemental digital content 5. Clinicopathological characteristics of all the pediatric patients for ten years.

| Variable | Mean (SD) or N (%) |
|---|---|
| Age | |
| 1 | 4,670 (51.9) |
| 2-5 | 2,324 (25.8) |
| 6-9 | 606 (6.7) |
| 10-13 | 532 (5.9) |
| 14-17 | 860 (9.6) |
| Sex, male | 5,086 (56.6) |
| Residence at ICPR | |
| Urban area | 3,746 (41.7) |
| Rural area | 5,246 (58.3) |
| Household income level | |
| Q1 | 1,527 (17.0) |
| Q2 | 1,669 (18.6) |
| Q3 | 2,993 (33.3) |
| Q4 | 2,425 (27.0) |
| Unknown | 378 (4.2) |
| Underlying congenital malformation | 2,392 (26.6) |
| Duration of ICPR | |
| <15 min | 3,585 (39.9) |
| 15-30 | 1,617 (18.0) |
| 30-45 | 1,131 (12.6) |
| 45-60 | 913 (10.2) |
| >60 | 1,502 (16.7) |

| | |
|---|---|
| Unknown | 244 (2.7) |
| LOS at ICPR | 19.1 (21.5) |
| Total cost for hospitalization at ICPR, USD | 16049.0 (20,888.3) |
| Insurance coverage | 15,540.9 (20,357.9) |
| Result of treatment | |
| Discharge and same hospital follow up | 1,922 (21.4) |
| Transfer to long-term facility care center | 243 (2.7) |
| Death within hospitalization after ICPR | 5,032 (56.0) |
| Discharge, and other outpt clinic follow up | 1,795 (20.0) |
| Year of ICPR | |
| 2010 | 806 (9.0) |
| 2011 | 862 (9.6) |
| 2012 | 886 (9.9) |
| 2013 | 820 (9.1) |
| 2014 | 830 (9.2) |
| 2015 | 827 (9.2) |
| 2016 | 1,069 (11.9) |
| 2017 | 1,019 (11.3) |
| 2018 | 960 (10.7) |
| 2019 | 913 (10.2) |

SD, standard deviation; ICPR, in-hospital cardiopulmonary resuscitation; LOS, length of hospitalization; USD, United States Dollar
